# Eculizumab Versus Ravulizumab for the Treatment of Atypical Hemolytic Uremic Syndrome: A Systematic Review

**DOI:** 10.7759/cureus.46185

**Published:** 2023-09-29

**Authors:** Kamran Shahid, Shahid Qayyum

**Affiliations:** 1 Internal Medicine/Family Medicine, California Institute of Behavioral Neurosciences & Psychology, Fairfield, USA; 2 Nephrology, Diaverum Dialysis Center, Wadi Al Dawasir, SAU

**Keywords:** genetic mutations, biomarkers, eculizumab, ravulizumab, atypical hemolytic uremic syndrome

## Abstract

Atypical hemolytic uremic syndrome (aHUS) is a type of thrombotic microangiopathy and is characterized by microangiopathic hemolytic anemia, thrombocytopenia, and acute kidney failure. The complement cascade plays an integral role in aHUS. Mutations in the complement cascade, especially in the alternative pathway (AP) lead to an unregulated and continuous activation of the cascade. Eculizumab and ravulizumab are humanized monoclonal antibodies that inhibit the complement cascade. This systematic analysis reviews the evidence for both antibodies to compare them in terms of safety and efficacy. This review will also assess the evidence for biomarker associations with interventions, the role of genetic mutations in the prognosis of disease, and the financial burden of both treatment options. An in-depth search was conducted across PubMed, Science Direct, and Cochrane Library following the PRISMA 2020 guidelines. Both eculizumab and ravulizumab were comparable in safety and efficacy but ravulizumab was preferred by patients and their caregivers as it posed a lower financial burden and had less frequent dosing. Soluble complement 5b-9 (sC5b), especially in urine, has the potential to be used as a biomarker to assess response to treatment. Genetic mutations, especially mutations in complement factor I (CFI), membrane cofactor protein (MCP), and complement factor H (CFH), were associated with a higher risk of recurrence, and therefore care should be taken when attempting to discontinue treatment in this subset of patients. Treatment with a monoclonal antibody should be initiated as soon as a genetic mutation is identified. Blinded, double-arm, clinical trials preferably with larger sample sizes are needed to effectively compare both the monoclonal antibodies.

## Introduction and background

Hemolytic uremic syndrome (HUS), most commonly caused by Shiga toxin-producing Escherichia coli O157:H7, is characterized by a triad of microangiopathic hemolytic anemia, thrombocytopenia, and acute kidney failure [1]. Atypical HUS (aHUS) is a variant of HUS that is not associated with Shiga toxin and the diagnosis is based on the presence of the triad as well as the exclusion of other forms of HUS or thrombotic thrombocytopenic purpura (TTP) [2]. The principal pathology of aHUS is capillary thrombosis which can lead to glomerular capillary occlusion resulting in renal injury. aHUS can also present with extrarenal manifestations in the central nervous, respiratory, gastrointestinal, and cardiovascular systems [3]. aHUS has an incidence of 0.23 to 0.42 cases/million/year with a mortality rate of up to 25% during the acute phase. Around 50% of the patients that survive the acute phase require acute renal replacement therapy (RRT) which in most cases results in a chronic need for RRT [4].

**Figure 1:**
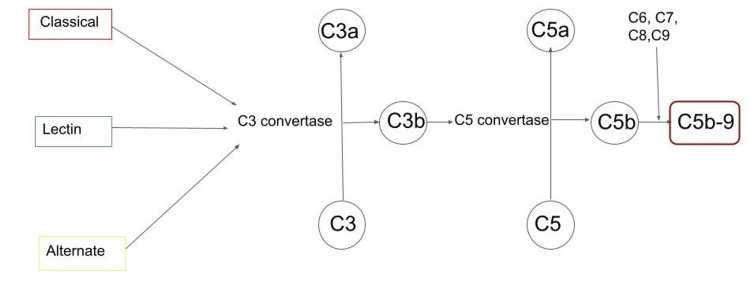
Simplified model of the complement cascade C= Complement component.

The complement system, once thought to play a supportive role in immunity, is now seen as a complex surveillance system that helps differentiate host cells from pathogens [5]. The complement system consists of three pathways: classical, lectin, and alternative. As shown in Figure 1, all three pathways lead to the activation of central complement component 3 (C3) which is cleaved by C3 convertase, an enzyme produced by the three pathways. All the pathways lead to the formation of complement component 5 (C5) convertase which cleaves C5 into C5a and C5b. The active C5b along with C6, C7, C8, and C9 form a membrane attack complex that plays a crucial role in the immune system [6-7].

With an increase in the understanding of the complement cascade, different mutations have been identified that play a role in aHUS with most of them being in the alternative pathway (AP) [8]. A dysregulation in the alternative complement pathway can lead to a continuous cleavage of C5 resulting in uncontrolled activation of the complement cascade [9]. This uncontrolled complement activation can be caused by the loss-of-function variants in regulatory proteins or gain-of-function variants in activation factors [10] such as complement factor H (CFH), a key regulator of the alternative pathway that inhibits complement activation and so genetic variants and autoantibodies against CFH can result in the development of aHUS [11]. Additionally, C5a possesses proinflammatory effects and is hypothesized to be a link between the inflammatory and thrombotic pathways [12].

The mainstay of treatment for aHUS used to be plasma exchange and plasma infusion which significantly decreased the mortality rate but a majority of the patients either were unable to tolerate regular therapy or relapsed upon cessation of the therapy. Liver transplantation either alone or accompanying renal transplant was used for some patients with inherited complement deficiencies but this intervention carries a higher risk of mortality. Two humanized monoclonal antibodies, eculizumab and ravulizumab, target C5 and inhibit the uncontrolled activation of the complement cascade [4].

This systematic review aims to compare eculizumab and ravulizumab in terms of their safety and efficacy in patients with aHUS. This review will also discuss the biomarker associations with intervention and the role of genetic mutation in the prognosis of the disease to further increase the understanding of this rare disease while also assessing the financial burden and impact on life caused by treatment.

## Review

Methodology

We performed a systematic evaluation using PubMed, ScienceDirect, and Cochrane Library and followed the Preferred Reporting Items for Systematic Reviews and Meta-Analyses (PRISMA) criteria.

Search Strategy

Full-text publications, free and paid, indexed in PubMed, ScienceDirect, and Cochrane Library were searched from inception to 2023 using the keywords “Atypical Hemolytic Uremic Syndrome”, “Eculizumab” and “Ravulizumab”. After the search was completed, duplicates were removed and abstracts and titles were used to identify relevant publications. The medical subject heading (MeSH) strategy used was ("Atypical Hemolytic Uremic Syndrome/therapy"[Majr]) AND ( "Antibodies, Monoclonal, Humanized/adverse effects"[Majr] OR "Antibodies, Monoclonal, Humanized/economics"[Majr] OR "Antibodies, Monoclonal, Humanized/therapeutic use"[Majr] ).

Eligibility Criteria and Study Selection

Eligibility was assessed by reading the full content of the publications. We selected the articles published from 2015-2023. Articles were only included if they were published in English or English translation was available. Articles were excluded if the full text of the papers could not be retrieved. Gray literature and studies done on animal subjects were excluded along with studies that included other types of TMA. Articles with only the pediatric population were also excluded.

Data Management

Articles were evaluated based on titles and abstracts, followed by a full-text examination. The data was cross-examined by both authors and any disputes were resolved. The first author's name, year of publication, study design, and the primary aim of the study were extracted. A narrative synthesis was performed on the extracted data.

Quality Assessment

We used the Assessment of Multiple Systematic Reviews (AMSTAR) form, the Cochrane risk-of-bias (RoB), and the Newcastle-Ottawa Scale for quality appraisal of the included studies.

Results

Search Results

**Figure 2:**
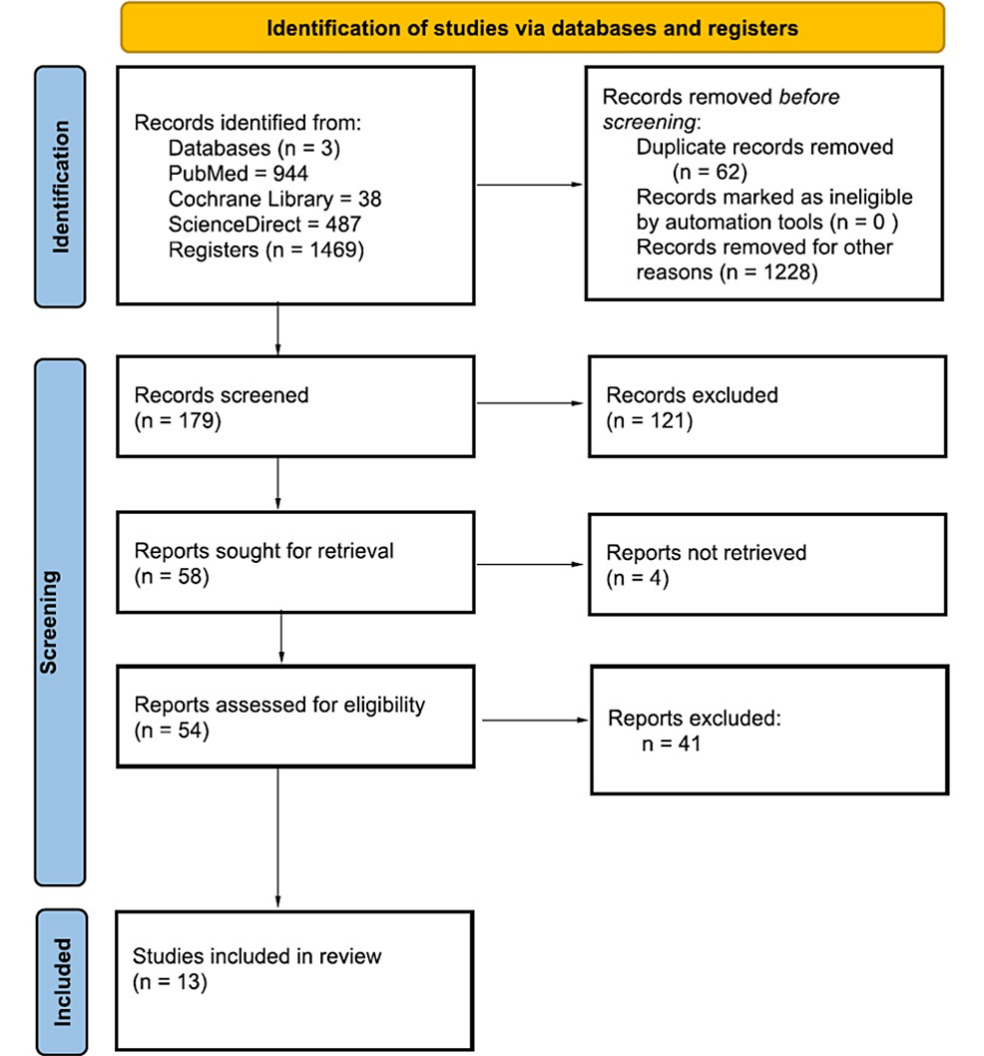
PRISMA flowchart of the literature and the search strategy

A total of 1469 studies were found after searching PubMed, ScienceDirect, and the Cochrane Library. A total of 1228 studies were removed as they did not meet the inclusion criteria. A further 62 articles were removed as they were duplicates. The remaining 179 articles were screened using full text of which 58 were sought for retrieval. As four articles could not be retrieved, 54 articles were assessed for eligibility of which only 13 were included in this review. Figure 2 shows the PRISMA flowchart of the literature and the search strategy of the studies [13].

Table 1 shows a summary and characteristics of all included studies.

**Table 1:** Characteristics of included studies aHUS= Atypical hemolytic uremic syndrome

Author (year)	Study design	The primary aim of the study
Legendre et al. (2017) [14]	Analysis of data pooled from four phase II clinical studies	To assess the efficacy and safety of eculizumab in patients with aHUS
Walle et al. (2016) [15]	Analysis of data pooled from four phase II clinical studies	To evaluate the importance of the time period between the onset of aHUS and treatment with eculizumab on renal and hematological outcomes
Zuber et al. (2019) [16]	Retrospective multicenter	Divided into two separate studies: Study one included patients with aHUS who underwent transplantation to assess the rate of recurrence; Study two assessed the fraction of the study population with aHUS who underwent dialysis or transplantation in association with eculizumab exposure
Rondeau et al. (2020) [17]	Single arm, global phase III trial	To assess the safety and efficacy of ravulizumab in adult patients with aHUS
Gäckler et al. (2021) [18]	Secondary analysis of the 311 trial [17]	To assess the safety and efficacy of ravulizumab in patients with pregnancy-triggered aHUS
Barbour et al. (2021) [19]	Extension of the 311 trial [17]	To assess the long-term safety and efficacy of ravulizumab in patients with aHUS
Tomazos et al. (2022) [20]	Analysis of data from clinical trials	To compare efficacy between eculizumab and ravulizumab from data available from clinical trials
Cofiell et al. (2015) [21]	Exploratory study of an open-label, nonrandomized, single-group, multicenter, trial	To assess the effects of eculizumab on patient biomarker levels
Cammett et al. (2022) [22]	Exploratory analysis of phase III clinical trial	To identify a set of biomarkers for prognosis and treatment response in aHUS
Krishnappa et al. (2017) [23]	Meta-analysis of case reports	Improve understanding of the efficacy of inhibiting the complement pathway in the treatment of aHUS and show a link to genetic mutations involved
Fakhouri et al. (2021) [24]	Open-label multicenter phase IV study	To study the safety and viability of eculizumab discontinuation
Mauch et al. (2023) [25]	Survey-based study	To assess preference for and treatment impact of ravulizumab versus eculizumab
Levy et al. (2022) [26]	Cross-sectional study	To assess the economic effect of ravulizumab and eculizumab in patients with aHUS

Discussion

Safety and Efficacy of Monoclonal Antibodies

**Figure 3:**
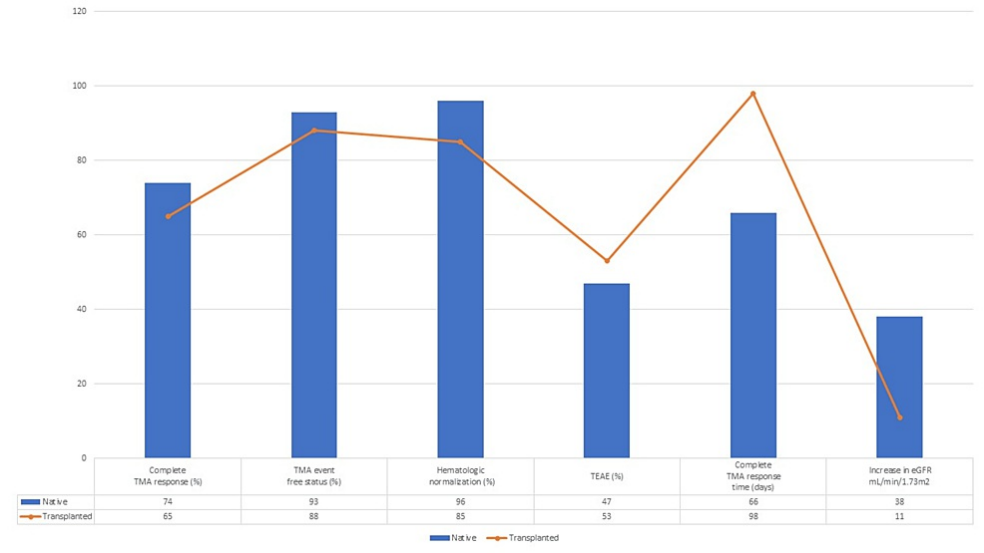
Safety and efficacy outcomes Legendre et al. [14] TMA= Thrombotic microangiopathy; TEAE= Treatment emergent adverse events; eGFR= Estimated glomerular filtration rate

Legendre et al. [14] was a post hoc analysis of comparing the outcomes with eculizumab treatment in patients with native and transplanted kidneys. Patients with transplanted kidneys showed fewer improvements in renal function and hematological normalization. Complete thrombotic microangiopathy (TMA) response defined as platelet count ≥150 x 109 /L, lactate dehydrogenase (LDH) levels less than the upper limit of normal, and ≥25% decrease in serum creatinine from baseline was reached by 65% of patients with transplanted kidneys and 74% of patients with native kidneys. Overall, eculizumab was well tolerated and was associated with improvement in renal function and hematological parameters. Graphical data of this study is shown in Figure 3.

**Figure 4:**
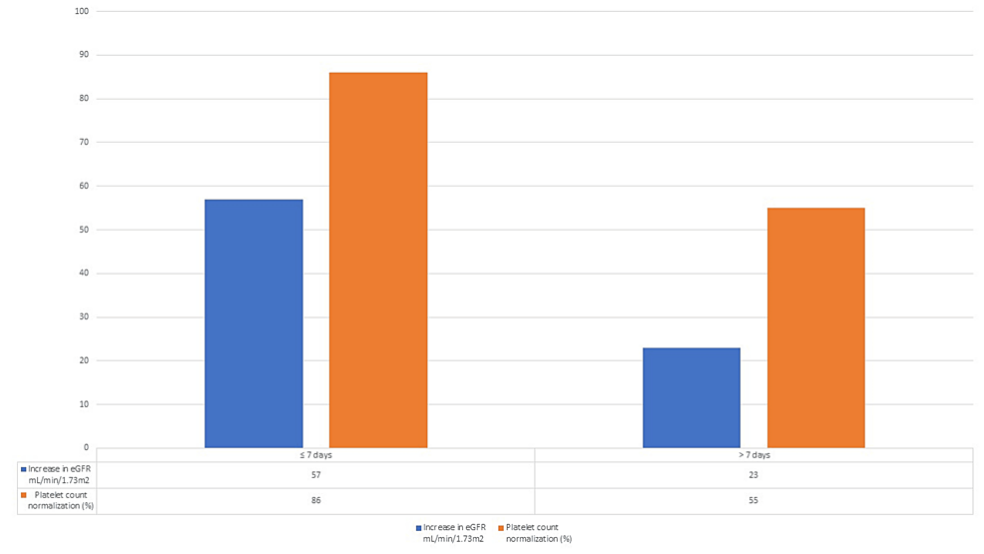
Graphical results of the study Walle et al. [15] eGFR= Estimated glomerular filtration rate

Another post hoc analysis conducted by Walle et al. [15] showed the significance of early identification and treatment initiation on the prognosis of patients with aHUS. Patients in whom the treatment with eculizumab was initiated in less than seven days after the manifestation of aHUS showed an increase of 57 mL/min/1.73m2 in estimated glomerular filtration rate (eGFR) and 86% of the patients attained platelet normalization. When treatment was started after seven days of manifestation there was a 23 mL/min/1.73m^2^ increase in eGFR and only 55% of patients achieved platelet normalization. Data from the study is shown in Figure 4.

Zuber et al. [16] a retrospective observational study on the use of eculizumab for prophylaxis against aHUS recurrence in kidney transplant recipients. Compared to plasma exchange, eculizumab therapy improved graft survival and was associated with a lower recurrence rate showing the usefulness of eculizumab as a prophylactic agent against aHUS. A total of 39 patients (52.7%) who were not given prophylaxis experienced aHUS recurrence compared to only four (7.7%) of the patients in the eculizumab group. Of the four patients in the eculizumab group who experienced recurrence, one patient discontinued after eculizumab was administered.

**Figure 5:**
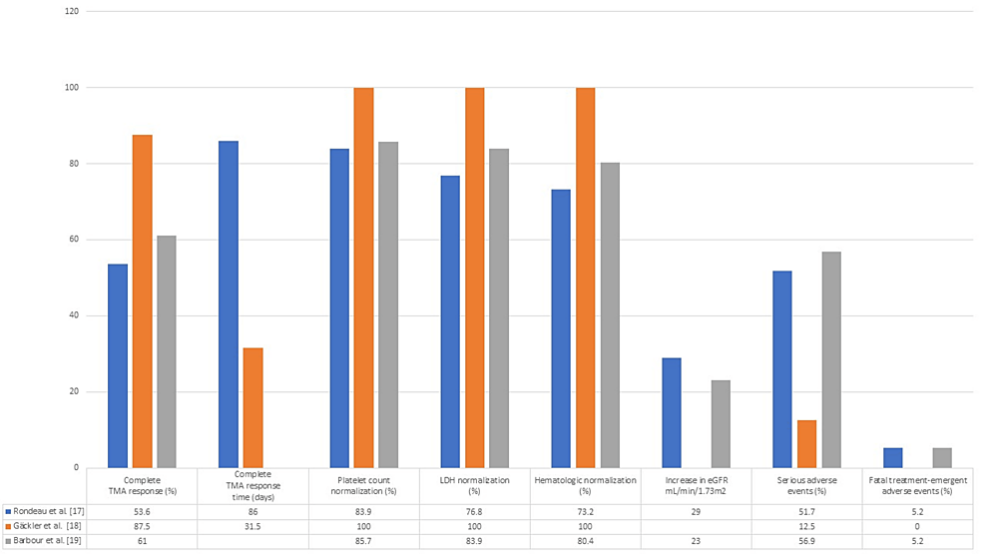
Safety and efficacy data of Ravulizumab TMA= Thrombotic microangiopathy; LDH; Lactate dehydrogenase; eGFR= Estimated glomerular filtration rate.

A phase III trial conducted by Rondeau et al. [17] found ravulizumab to be effective for the management of aHUS with 53.6% of the patients reaching a complete TMA response with the median time to complete response of 86 days. There was an increase in eGFR of ≥ 15 mL/min/1.73m^2^ in 68.1% of the included patients. There were no unexpected adverse events but 51.7% of the patients experienced serious adverse events and 5.2% of patients experienced fatal treatment-emergent adverse events. One benefit of ravulizumab is the low dosing frequency limited to once every eight weeks, which reduces the treatment burden on the patients and their families. Gäckler et al. [18] analyzed the 311 trial [17] focusing on a subgroup of patients with postpartum aHUS. All the patients in this subgroup presented with severe aHUS. The median time to complete TMA response was 31.5 days with 87.5% of patients reaching complete TMA response. The one patient who did not reach the primary endpoint showed a rapid response to ravulizumab and achieved platelet and LDH normalization by Day 8. The patients in this subgroup received the first dose earlier (median of 11 days) as compared to the original trial signifying the impact of early initiation of treatment. Barbour et al. [19] conducted an extension of the 311 trial [17] to evaluate the long-term safety and efficacy of ravulizumab in patients who had completed the 311 trial. The median follow-up period for this study was 76.7 weeks. At Week 26, 53.6% of patients had reached complete TMA response in the 311 trial. During the extension, complete TMA response was attained by four more patients with a total of 61% of patients who had now attained the criteria for complete TMA response. There were further improvements in LDH normalization (83.9% from 76.8%), platelet count normalization (85.7% from 83.9%), and hematological normalization (80.4% from 73.2%) during the extension phase. Ravulizumab was well tolerated with no unexpected adverse events with 56.9% of the patients experiencing serious adverse effects and 34.5% of patients experiencing treatment-related adverse events. Graphical data from all three studies is shown in Figure 5.

Tomazos et al. [20] conducted an indirect comparison of efficacy between eculizumab and ravulizumab using data from clinical trials. This trial found that at Week 26, a higher percentage of patients were undergoing dialysis in the ravulizumab group than in the eculizumab group (22% vs. 8%) but the mean eGFR values favored ravulizumab (55.4 mL/min/1.73m^2^ vs 51.4 mL/min/1.73m^2^). Complete TMA response was reached by 70% of the patients in the eculizumab group and 61% in the ravulizumab group. The author concluded that there was no statistical difference in efficacy between ravulizumab and eculizumab at Week 26 as they both provided comparable and significant improvements.

Biomarker Associations With Intervention

Cofiell et al. [21] conducted an exploratory study to assess the effects of eculizumab on patient biomarker levels. The biomarkers and their role in the disease process are shown in Table 2.

**Table 2:** Biomarkers and their role in disease process AP= Alternate pathway; C5= Complement component 5.

Biomarker	Disease process
Complement Factor Ba	Marker for AP activation
Complement component 5a	Marker for terminal complement (C5) activation
Soluble complement 5b-9 (sC5b-9)	Marker for terminal complement (C5) activation and forms MAC
Soluble tumor necrosis factor receptor-1	Marker for inflammation
Soluble vascular cell adhesion molecule-1	Marker for endothelial cell activation
Thrombomodulin	Marker for endothelial cell damage
Prothrombin fragment F1+2	Marker for thrombin generation (coagulation)
D-dimer	Marker for fibrinolysis (coagulation)
Urine cystatin-C, Clusterin, β2-microglobulin	Marker for renal injury (proximal tubule damage)
Tissue inhibitor of metalloproteinase (TIMP)-1	Marker for renal injury interstitial tubule damage)
Liver fatty acid binding protein (L-FABP)-1	Marker for renal injury

At baseline, patients had elevated levels of all biomarkers. Levels of Ba remained elevated throughout the one-year treatment period signifying that there was continued AP activation upstream. Levels of C5a and soluble complement 5b-9 (sC5b) immediately dropped after treatment with eculizumab was initiated, showing complete downstream complement blockade by eculizumab. All biomarkers for renal injury became almost identical to that of healthy volunteers by Week 26 and there was a constant reduction in markers of coagulation as treatment progressed, showing that eculizumab treatment significantly reduced coagulation and renal damage. Inflammation in aHUS is the main contributor to endothelial activation and damage and treatment with eculizumab showed a reduction in soluble tumor necrosis factor receptor-1 (sTNFR1) and thrombomodulin levels but the levels of soluble vascular cell adhesion molecule-1 (sVCAM-1) remained elevated after one year of treatment. This study showed that treatment with eculizumab reduces inflammation, endothelial damage and activation, and coagulation but there is continued upstream AP activation signified by sustained levels of Ba and sVCAM-1 placing patients at life-long risk of TMA. 

Cammett et al. [22] conducted an exploratory analysis to identify biomarkers that may show usefulness in the management of aHUS with ravulizumab. Elevated levels of factor Ba and sC5b-9 were found in more than 85% of patients and showed a significant association with renal dysfunction before treatment initiation. At baseline, the patient's pretreatment plasma exchange/plasma Infusion and dialysis status showed associations with biomarker levels. Patients who underwent plasma exchange/plasma infusion within seven days of treatment initiation showed lower levels of plasma sC5b-9 levels and patients who underwent dialysis within five days showed higher levels of plasma Ba, serum sTNFRI, and urine Ba/creatinine. A lower eGFR at baseline was associated with higher levels of plasma Ba, plasma thrombomodulin, serum sTNFRI, urine cystatin-C/creatinine, sC5b-9, and Ba/creatinine. Additionally, urine, as a matrix showed to be of higher clinical significance as urine sC5b-9 levels, showed significant associations with change in renal function while plasma sC5b-9 showed lesser associations with clinical outcomes. This study concluded that use of urine sC5b-9 could be used as a potential biomarker for aHUS.

Role of Genetic Mutations in the Prognosis of aHUS

Mutations in CFH, complement factor I (CFI), complement factor B (CFB), complement factor H-related protein 1 (CFHR-1), and membrane cofactor protein (MCP) have been identified to play a role in aHUS. CFH gene mutations and anti-CFH autoantibodies disrupt the function of CFH and lead to thrombotic microangiopathy predominantly in the glomerular capillary bed. Of the patients screened for CFH mutations, 50% were positive. CFI, in the presence of its cofactor MCP, plays a role in the downregulation of the alternative and classic complement pathways. CFI and MCP mutations were found in 16.6% and 22.8% of patients tested respectively. Patients with CFI and CFH mutations carried a higher risk of recurrence and CFH mutation was associated with poor outcome. Patients with mutations, who were treated with plasma exchange had a higher mortality and a longer time to resolution of symptoms showing that genotype plays a significant role in the outcome and that treatment with eculizumab should be started as soon as genetic mutations are identified [23].

The retrospective study by Zuber et al. [16] showed that carriers of the MCP mutation had a risk of recurrence that was not affected by eculizumab prophylaxis. The chances of recurrence in patients with MCP or diacylglycerol kinase epsilon (DGKE) were low and there was no need for prophylaxis against recurrence in this group of patients. Additionally, patients who did have mutations in CFH and CFI were at a reduced risk of recurrence. This study signified that the use of Eculizumab as prophylaxis against recurrence should be based on the genotype of the patient.

Fakhouri et al. [24] conducted a prospective study to assess the viability of Eculizumab discontinuation. The study found that the risk of relapse after discontinuation was higher in patients with complement mutations, especially in the CFH and MCP genes. Additionally, female sex and elevated sC5b-9 level at the time of Eculizumab discontinuation are associated with a higher risk of relapse. Therefore, Eculizumab discontinuation for patients with no identified complement mutations is a viable strategy as it improves the quality of life of the patient and reduces the cost of treatment. However, if patients with no identifiable complement variants do relapse, it should prompt a reevaluation of the genetic testing.

Quality of Life Impact and Financial Burden With Interventions

Mauch et al. [25] conducted a study using web-based surveys to assess the impact of intervention used on the quality of life and the preference for a particular drug. Among adults, 4% and 5.7% of the respondents reported that ravulizumab impacted their daily lives and their ability to go to work/school compared to a staggering 72% and 60% for eculizumab. Furthermore, 94% of adult patients and all caregivers showed a preference for ravulizumab. This could be attributed to the dosing frequency of ravulizumab (once every four to eight weeks) and eculizumab (once every two weeks). Furthermore, 94% of adult patients and all caregivers reported a preference for ravulizumab.

According to Levy et al. [26] the total duration of treatment administration for 100 patients over one year for 100 mg/mL ravulizumab ranged from 3,558 to 6,664 hours, for 10 mg/mL ravulizumab from 7,291 to 10,895 hours, and for 10 mg/mL eculizumab from 13,873 to 21,870 hours. In terms of the cost of annual treatment, treatment with ravulizumab 100 mg/ml had the lowest total cost. Compared to eculizumab, ravulizumab 100mg/ml resulted in a total saving of 73% in the annual lost productivity costs (calculated assuming a pay of $20 an hour and the total work hours lost due to treatment per year).

Limitations

This review has certain limitations. Firstly, the review includes single‐arm studies, which can introduce bias in the results. Secondly, there are no head-to-head clinical trials comparing eculizumab and ravulizumab. Thirdly, the sample size of most of the included trials was small, something which is expected for a rare disease that limits the detection of differences between treatments. Lastly, the study used to estimate the financial burden was based on assumptions made on the level of support given by caregivers and on a hypothetical population of patients with aHUS.

## Conclusions

aHUS is a rare disease characterized by a triad of microangiopathic hemolytic anemia, thrombocytopenia, and acute kidney failure caused by a dysfunctional complement cascade. Eculizumab and ravulizumab are two humanized, monoclonal antibodies used for the treatment of aHUS. Both antibodies are well tolerated and have comparable efficacies in improving renal function, hematological markers, and dialysis prevalence. Biomarkers such as sC5b-9, especially in urine have the potential to be used to assess the efficacy of the treatment. Genetic mutations such as mutations in CFH, CFI, CFB, CFHR-1, and MCP play a significant role in the prognosis of the disease. Mutations in CFI, CFH, and MCP are associated with a higher risk of recurrence. While ravulizumab and eculizumab are comparable in safety and efficacy, ravulizumab was preferred by a majority of the patients as it only has to be dosed once every eight weeks. This led to a decreased financial burden and a better quality of life for both the patients and their caregivers. Blinded, double-arm, clinical trials preferably with larger sample sizes are needed to effectively compare both the monoclonal antibodies.
